# Gender- and stressor-specific microRNA expression in *Tribolium castaneum*

**DOI:** 10.1098/rsbl.2012.0273

**Published:** 2012-05-23

**Authors:** Dalial Freitak, Eileen Knorr, Heiko Vogel, Andreas Vilcinskas

**Affiliations:** 1 Institute for Phytopathology and Applied Entomology, Justus Liebig University of Giessen, Heinrich Buff Ring 26-32, 35392 Giessen, Germany; 2 Entomology Department, Max Planck Institute for Chemical Ecology, Hans Knoell Strasse 8, 07749, Jena, Germany

**Keywords:** *Tribolium castaneum*, microRNA, gender studies, epigenetic, immunity, environmental stress

## Abstract

MicroRNAs (miRNAs) are small non-coding RNAs mediating post-transcriptional regulation of gene expression in eukaryotes. Addressing their role in regulation of physiological adaptations to environmental stress in insects, we selected the red flour beetle *Tribolium castaneum* as a model. Beetles were fed with the bacterial entomopathogen *Pseudomonas entomophila* (to mimic natural infection), injected with peptidoglycan (experimental setting of strong immune responses) or subjected to either mild heat shock or starvation. Differential expression of selected immunity- and stress-related genes was quantified using real-time PCR, and expression and induction of 455 mature arthropod miRNAs were determined using proprietary microarrays. We found that *Tribolium* exhibits both gender- and stressor-specific adjustment of immune gene and miRNA expression. Strikingly, we discovered that the number of stressor-induced miRNAs in females is remarkably higher than in males. This observation could support the hypothesis called Bateman's principle in immunity that predicts gender-specific immune responses because females gain fitness through increased longevity, whereas males gain fitness by increasing mating rates. Our results suggest that *Tribolium* males and females display differential regulatory elements, both pre- and post-transcriptional, likely resulting from different investment strategies in life-history traits.

## 1. Introduction

MicroRNAs (miRNAs) are small approximately 22-nucleotide (nt) long non-coding RNA molecules generated from stem–loop hairpin structures called miRNA precursors. [1]. Their main function is the downregulation of gene expression by base-pairing with the 3′ untranslated regions of target messenger RNAs. Hundreds of miRNAs have been identified since the discovery of the first members 20 years ago [2]. However, most identified miRNAs are computational predictions, lacking experimental confirmation of their expression. A single miRNA can regulate hundreds of different target genes and more than 30 per cent of the genes in animals are believed to be under control of miRNAs [2,3]. Consequently, they determine the phenotype of an organism at the post-transcriptional and translational level. Whether the entity of miRNAs may constitute a fine-scale regulatory mechanism underlying physiological responses to environmental changes is poorly understood and has therefore been addressed here. We selected the red flour beetle *Tribolium castaneum* to explore whether miRNAs contribute to the regulation of immune and stress responses, and whether their regulation by miRNAs may explain gender-specific differences.

To enable quantification of immune or stress responses, we determined expression rates of typical effector genes contributing either to immune (three antibacterial defensins and the antifungal protein thaumatin) or stress responses (a CYP450 protein and three heat shock proteins) [4,5]. Expression profiling of the selected eight genes was used to monitor either immune or stress responses in beetles upon exposure to environmental stress imposed by microbial challenge, starvation or mild heat shock. The previously observed simultaneous induction of antimicrobial peptides and heat shock proteins in *Tribolium* implicates a crosstalk between immune and stress responses [4–6]. Beetles were fed with the bacterial entomopathogen *Pseudomonas entomophila* (PE) to mimic natural infection because oral uptake of bacteria is reportedly sufficient to activate systemic immune responses associated with fitness costs in insects [7]. We monitored induced RNA and miRNA expression in response to either fed bacteria or injected peptidoglycan (PGN) because the latter is known for its high capacity to induce immune responses. Further, we subjected the beetles either to mild heat shock or to starvation. Using miRNA sequences available in the public miRBase (http://www.mirbase.org/) v. 14 and those from literature [1], we developed an arthropod-specific microarray containing 455 unique mature miRNA sequences based on the hypothesis that the majority of mature miRNA sequences is conserved among arthropods [8].

Beyond differential expression of selected miRNAs and RNAs upon exposure to environmental stressors we were interested to elucidate whether the latter results in gender-specific effects because sex-specific life histories could select for investment in immune and stress responses. The limited number of studies providing experimental evidence for this hypothesis called Bateman's principle in immunity [9] calls for complementary research, in particular, studies addressing the underlying regulatory mechanisms [10,11]. Consequently, male and female beetles were separately subjected to the different stressors to allow assessment of gender-specific effects.

## 2. Material and methods

### (a) Animal breeding and treatment

Wild-type *T. castaneum* strain San Bernardino were reared on whole-grain flour with 5 per cent yeast powder at 32°C in darkness. Pupae were separated by gender, kept in separated Petri dishes and hatched adults were collected daily. Ten-day-old female and male *Tribolium* beetles were subjected to starvation, heat stress, PE feeding or PGN injection, with the control group consisting of animals fed on standard diet (more information in the electronic supplementary material).

### (b) Quantitative real-time PCR

Each experimental group consisted of 2 × 15 females and 2 × 15 males which were shock frozen in RNAlater (Ambion) and stored at −80°C until further analysis. Gene-specific primers were designed on the basis of sequences obtained for selected *Tribolium* genes and the 18S rRNA gene served as the endogenous control (normalizer). Quantitative real-time PCR was done in optical 96-well plates on a CFX 96 real-time PCR Detection System (BioRad) using the SsoFast EvaGreen Supermix (BioRad) (more information in the electronic supplementary material).

### (c) MicroRNA reference sets and microRNA microarray assay

MicroRNA sequence data used here originate from the miRNA Registry Database (Release 14; http://microrna.sanger.ac.uk), with (i) complete miRNA sequences characterized or predicted for arthropods; and (ii) the published, computationally predicted *Tribolium-*specific miRNAs not added to the database yet. A recent study has expanded the number of *Tribolium* miRNAs using NextGen sequencing [8] but could not be included in the microarray generation process as it became available after our analysis. Proprietary miRNA microarray assays were done using a service provider (LC Sciences, Houston, USA) (see electronic supplementary material). The assays were done on 5 µg of total RNA samples from two biological replicates (each replicate consisted of pooled RNA sample originating from 15 animals) of *Tribolium* female and male adults from different treatments, where one biological replicate was labelled with Cy3 and the other with Cy5. Labelled samples were co-hybridized on one chip.

For each chip and each probe, the average signal value and its s.d. were quantified. Data were analysed by first subtracting the background, and then integrating all the signals corresponding for the same probe for one given chip. A transcript to be listed as detectable must meet at least two conditions: signal intensity higher than three times (background s.d.) and spot CV < 0.5. Since repeating probes are present on an array, a transcript is listed as detectable only if the signals from at least 50 per cent of the repeating probes are above detection level. Normalization of the signals from all arrays was performed using a LOWESS filter (Locally-weighted Regression). Intensity values were transformed into log2 scale, and fold changes were given in log2 scale. Results obtained in the different treatments were analysed by comparing the ratio of the two sets of detected signals (log2) and *p*-values of the *t*-test were calculated. Differentially detected signals were defined as those with a *p*-value < 0.05. Data classification involved a hierarchical clustering method using average linkage and Euclidean distance metric, and was visualized with TIGR's MeV (Multiple Experimental Viewer; Institute for Genomic Research). The obtained microarray data were verified by expression profiling of several miRNAs using RT-qPCR (more information in the electronic supplementary material).

## 3. Results

### (a) Expression of immune- and stress-related genes

**Figure 1.**
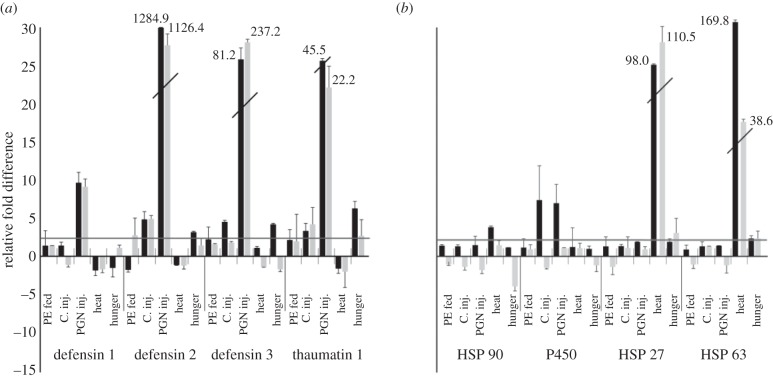
Relative fold difference (±s.d.) in immune gene expression induced upon treatment with bacteria, heat shock or starvation. (*a*) Immune response-related genes, comparison between females and males and (*b*) stress response-related genes, comparison between females (black bars) and males (grey bars). Treatments: *Pseudomonas entomophila* fed (PE fed), sham injection with saline solution (C. inj.), peptidoglycan injection (PGN inj.), heat shock (heat) and starvation (hunger). Only induced levels above the twofold of the controls (labelled with a bar) were considered.

Our real-time PCR-based comparative analysis of selected immune- and stress-related genes elucidates differential- and gender-specific patterns of induced expression levels in response to microbial challenge, heat shock or starvation. As previously reported [6,7], we observed that injected PGN induced remarkably stronger immune responses than oral administration of bacteria (figure 1). All three defensins and thaumatin reached by far the highest induced expression levels. As expected, we observed high induction of heat shock proteins in response to mild heat shock. Interestingly, we determined evident gender-specific differences in stress-induced expression levels among the selected genes. For example, P450 expression upon injection of microbial elicitors was significantly upregulated in females, but not in males. Unexpectedly, we observed that starvation results in induced expression of defensin 3 and thaumatin (figure 1). Subsequently, we studied whether the determined differential- and gender-specific expression pattern of selected immunity and stress-responsive genes could be regulated by differential expression of miRNAs.

### (b) Stress-induced expression of microRNAs

To compensate for the lacking information about the *in vivo* expression of the increasing number of miRNAs predicted from different datasets, we designed an array combining 386 already confirmed and 69 predicted miRNA sequences. Qualitative and quantitative detection of selected miRNAs revealed that at least seven of these are highly expressed upon treatment with bacteria, heat shock and starvation in both genders (electronic supplementary material, table S1 and figure S1). Interestingly, oral uptake of PE resulted in downregulation of 11 miRNAs, while only three were upregulated (*p* < 0.05; bmo-miR-190, C_tca-miR-516* and dpu–miR-258). In line with the observed strong induction of antimicrobial peptides upon injection of PGN, we determined 59 miRNAs to be differentially expressed upon challenge among which 21 were upregulated. Exposure of *Tribolium* to mild heat shock or starvation induced only relatively few miRNAs (six upon heat shock and five in response to starvation) when compared with the high number of downregulated miRNAs (40 upon heat shock and 22 in response to starvation) (electronic supplementary material, table S1).

For the confirmation of microarray results, we analysed expression levels of seven miRNAs with RT-qPCR. To correct for sample-to-sample variation, we selected three miRNAs with constant expression as endogenous controls (reference miRNAs) based on our microarray data. All three reference miRNAs displayed uniform expression across all samples in RT-qPCR assays, confirming our microarray data. Furthermore, the expression of all but one miRNA analysed by RT-qPCR matched microarray expression data.

### (c) Gender-specific expression of microRNAs

**Figure 2.**
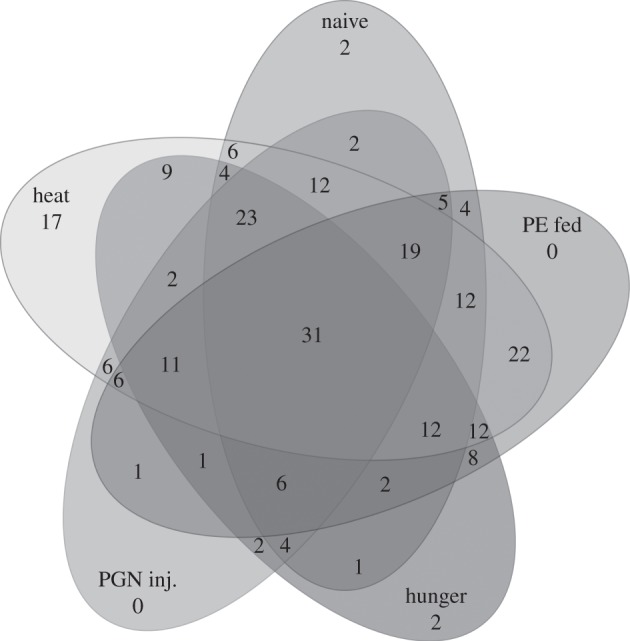
Gender-dependent differential expression of miRNAs. The Venn diagram shows the number of shared and unique miRNAs expressed upon different stressor treatments. In total, 245 miRNAs showed stress- and gender-dependent expression. All of the miRNAs shown here display more than twofold expression differences between the genders (*p* < 0.05). The ratio of signal intensities and *p*-values of individual miRNAs have been summarized in electronic supplementary material, table S1.

Strikingly, a total of 245 miRNAs (i.e. 54%) exhibited gender-specific expression patterns upon exposure to environmental stress. The percentage of gender specifically expressed miRNAs ranged between 47 per cent (upon starvation) and 28 per cent (upon PGN injection; table 1). 31 miRNAs showed genders specific expression upon all treatments (figure 2 and electronic supplementary material, table S1). Eleven miRNAs were differentially expressed even in untreated male and female beetles, among which 7 exhibited higher transcript levels in females and four in males, respectively (electronic supplementary material, table S1). A two-factorial ANOVA analysis confirmed gender- and stressor-specific expression of miRNAs in *Tribolium* (electronic supplementary material, table S2).

**Table 1.** Gender specific expression of miRNAs upon different treatments (% of total miRNAs). In total 54 per cent of miRNAs showed stress and gender specific expression.

treatment	in total	up in ♀	up in ♂
naive	32	31	1
heat	47	34	13
hunger	29	27	2
PGN inj.	28	27	1
PE fed	33	32	1

## 4. Discussion

The differential expression of the selected immune- and stress-related genes used to determine the efficacy of our induction regime is in line with previous studies [6]. Highest induction levels were recorded for the antimicrobial peptides upon injection of PGN, which is known for its high potency to elicit innate immune responses, whereas highest expression levels of the heat shock proteins occurred upon mild heat shock. The unexpected induction of defensin 3 and thaumatin in response to starvation is in agreement with the recently discovered expression of antimicrobial peptides in food-deprived *Drosophila melanogaster.* The nuclear transcription factor FOXO, a key regulator of stress resistance, metabolism and ageing, has been attributed to mediate AMP expression in response to starvation in this model insect [12]. The determined induction of CYP450 in female *Tribolium* reflects solely a gender-specific response to injected PGN which can be explained by the need to protect developing eggs from toxins associated with pathogens. A similar female-specific investment in offspring explains the higher expression of HSP63 and HSP90 in female beetles upon mild heat shock (figure 1). At least HSP90 is known to function as a chaperon and to play a role in oogenesis [5]. In sum, our real-time PCR-based approach provides evidence for differential- and gender-specific expression of immune- and stress-related genes upon exposure to environmental stressors. Since it has been established that miRNAs can target genes for translational inhibition or mRNA degradation [13], we expected also diverse expression patterns for the selected miRNAs. To fill the growing gap between the increasing number of predicted miRNAs added to the global miRNA database and information about their expression and function [8], we investigated whether particular miRNAs contribute to regulation of genes which are differentially expressed in response to biotic and abiotic stress. Indeed, 54 per cent out of a total of 455 microRNAs displayed stressor-dependent differential- and/or gender-specific expression.

Based on our findings, it is intriguing to speculate whether the observation that immediate stress (such as heat) result in a larger fraction of regulated miRNAs, while longer-term stress (e.g. starvation) lead to a lower number of differentially expressed miRNAs, could be advantageous for plastic responses of *Tribolium* to environmental stress. This ultimately could also explain the remarkably higher number of miRNAs upregulated in females upon exposure to stress when compared with males (table 1). This discovered discrepancy in gender-specific miRNA expression could support the hypothesis called Batemańs principle in immunity [9] because males gain fitness by increasing their mating success whereas females increase fitness through longevity since their reproductive effort is higher [10,11]. Consequently, their ability to cope with environmental stressors should be higher than in males and this is reflected by the determined broader spectrum of miRNAs involved in regulation of immune and/or stress responses. While the precise function of individual miRNAs remains to be elucidated, our study provides for the first time information about miRNAs in *T. castaneum* exhibiting differential- and gender-specific expression pattern upon exposure to different stressors. Whether these changes translate into environmentally induced heritable epigenetic changes [14] remains to be elucidated.
